# Divergence Times and Phylogenetic Patterns of Sebacinales, a Highly Diverse and Widespread Fungal Lineage

**DOI:** 10.1371/journal.pone.0149531

**Published:** 2016-03-03

**Authors:** Sigisfredo Garnica, Kai Riess, Max E. Schön, Franz Oberwinkler, Sabrina D. Setaro

**Affiliations:** 1 University of Tübingen, Institute of Evolution and Ecology, Plant Evolutionary Ecology, Auf der Morgenstelle 1, 72076, Tübingen, Germany; 2 Wake Forest University, Department of Biology, 205 Winston Hall, 1834 Wake Forest Road, Winston-Salem, North Carolina, 27106, United States of America; Earth and Life Institute, BELGIUM

## Abstract

Patterns of geographic distribution and composition of fungal communities are still poorly understood. Widespread occurrence in terrestrial ecosystems and the unique richness of interactions of Sebacinales with plants make them a target group to study evolutionary events in the light of nutritional lifestyle. We inferred diversity patterns, phylogenetic structures and divergence times of Sebacinales with respect to their nutritional lifestyles by integrating data from fossil-calibrated phylogenetic analyses. Relaxed molecular clock analyses indicated that Sebacinales originated late Permian within Basidiomycota, and their split into Sebacinaceae and Serendipitaceae *nom*. *prov*. likely occurred during the late Jurassic and the early Cretaceous, coinciding with major diversifications of land plants. In Sebacinaceae, diversification of species with ectomycorrhizal lifestyle presumably started during the Paleocene. Lineage radiations of the core group of ericoid and cavendishioid mycorrhizal Sebacinales started probably in the Eocene, coinciding with diversification events of their hosts. The diversification of Sebacinales with jungermannioid interactions started during the Oligocene, and occurred much later than the diversification of their hosts. Sebacinales communities associated either with ectomycorrhizal plants, achlorophyllous orchids, ericoid and cavendishioid Ericaceae or liverworts were phylogenetically clustered and globally distributed. Major Sebacinales lineage diversifications started after the continents had drifted apart. We also briefly discuss dispersal patterns of extant Sebacinales.

## Introduction

Geographic distributions of fungi on a large scale are strongly influenced by their respective lifestyles [1], dispersal limitation, surrounding plant communities, as well as by climate and habitat (see e.g. [2–4]). In general, saprobic fungi show a broader geographic distribution than biotrophic fungi, because the latter often have specific host requirements. The most important biotrophic association between fungi and plants are mycorrhizae [5]. Most mycorrhizal basidiomycetes form ectomycorrhiza, a type of mycorrhiza that evolved several times independently in plants and fungi. It can be found in about 80 different, mainly woody plant lineages and primarily in association with Basidiomycota, but also with Ascomycota [6]. Basidiomycota form various other mycorrhizal types, such as orchid, ericoid and arbutoid mycorrhiza. These types are mainly restricted to a single host plant family. Orchid mycorrhiza occurs only in orchids, ericoid mycorrhiza only in Ericaceae and ferns, and arbutoid mycorrhiza in early lineages of Ericaceae (*Arbutus*, *Arctostaphylos* and *Pyrola*) [5]. Cavendishioid mycorrhiza has been found mainly in neotropical Ericaceae belonging to the so-called Andean Clade [7]. In addition to intracellular colonization, cavendishioid mycorrhiza is characterized by a hyphal sheath as well as a Hartig net. Sebacinales forming cavendishioid mycorrhiza are closely related to ericoid forming Sebacinales and seem to be able to form both types [8].

In addition to mutual symbioses, many ascomycetes and some basidiomycetes form endophytic interactions with a broad range of plants [9]. The most diverse basidiomycete order in terms of mutualistic interactions is Sebacinales [10]. Sebacinales engage in a range of mycorrhizal interactions including ectomycorrhizal association with trees (e.g. [11]) and herbs [12, 13], mycorrhizal associations with Ericaceae (e.g. [14–16]), photoautotrophic orchids [17, 18] and heterotrophic orchids [19], and mycorrhiza-like interactions with liverworts [20, 21]. These fungi also occur inside herbaceous plants roots [22–25], presumably forming endophytic interactions. Some Sebacinales are not associated with plants and have a saprobic lifestyle. Most of this knowledge has been gathered during the last decade by means of rDNA sequencing of fungal material from environmental samples. Moreover, these studies also revealed that Sebacinales are a highly diverse group with a global geographic distribution and some degree of host specialisation. Sebacinales are molecularly distinguished into two groups, Sebacinaceae or known as Sebacinales Group A and Serendipitaceae *nom*. *prov*. currently named Sebacinales Group B [10, 26]. Sebacinaceae are mainly saprobic, ectomycorrhizal or involved in tripartite symbioses involving ectomycorrhizal plants, whereas Serendipitaceae mainly form orchid and ericoid mycorrhiza as well as mycorrhiza-like interactions with liverworts. Endophytic Sebacinales are found in both families. However, host specificities do not seem to occur at the species level or molecular operational taxonomic units (MOTUs) [24, 25, 27]. Little is known about the evolutionary history of Sebacinales with respect to geographic distribution, nutritional traits and plant hosts. Tedersoo and coauthors (2014) studied divergence times and biogeographic patterns of Sebacinaceae, but did not address Serendipitaceae. Their results suggested that Sebacinaceae are relatively young (45–58 million years ago [mya]) in comparison to other ectomycorrhizal groups and that affiliation with a host plant family had little effect on their evolutionary history.

In contrast to some ectomycorrhizal fungi [28–29], long distance dispersal events may explain some large-scale disjunct distributions of ectomycorrhizal Sebacinales [30]. However, only very few Sebacinales have been found to form fruiting bodies and all of these belong to Sebacinaceae. In Serendipitaceae, only *Serendipita vermifera* has been found to reproduce sexually but without the formation of a conspicuous fruiting body [17, 31]. Asexual spore formation has been reported for *Piriformospora indica*, *Serendipita herbamans* and *S*. *vermifera*, but it is unknown how frequent spores are formed in nature.

The relatively widespread and frequent occurrences of Sebacinales, their ability to establish a broad spectrum of different lifestyles occurring in most terrestrial ecosystems and their presumably early divergence within Basidiomycota [32] make them a model for further exploration of ecological specialisation and biogeographic history. In the present study, we combined nuclear and mitochondrial sequence data of representative species within the phylum Basidiomycota to infer a fossil-calibrated phylogeny and reconstruct divergence times for Sebacinales. In addition, we used internal transcribed spacer (ITS) sequence data to examine the effects of nutritional lifestyle on the community structure of Sebacinales as a whole. We addressed two main questions: (i) How does the divergence time of these strategies relate to the evolution of their respective hosts, and (ii) does the phylogenetic structure of Sebacinales correspond to nutritional lifestyle?

## Material and Methods

### Ethics statement

The fungal species used in this study were not protected and specimens were traded according to standard international herbaria policy and loan regulations.

### Taxon sampling

Our sampling approach involved three steps: (i) generating a sequence dataset with representatives of Basidiomycota using different genes (18S, 28S, *rpb*1, *atp*6) to estimate the relative age of the Sebacinales within Basidiomycota (S1 Table); (ii) compiling a dataset of Sebacinales spanning the ITS region to estimate the relative ages of the Sebacinales lineages (S1 Data); and (iii) examine large-scale patterns of the phylogenetic structures of Sebacinales communities using the ITS dataset.

### DNA isolation, PCR, cloning and sequencing

Total genomic DNA was extracted from dried basidioma fragments or cultures using the InnuPREP Plant DNA Kit (Analytik Jena, Jena, Germany) following the manufacturer’s instructions. Fungal portions were placed in Eppendorf tubes and deep-frozen in liquid nitrogen and ground with a sterile plastic pestle.

For Basidiomycota, including representatives of Sebacinaceae (*Craterocolla cerasi* and *Sebacina incrustans*) and Serendipitaceae (*Piriformospora indica* and *Serendipita* sp.), the 18S gene of the nuc-rDNA was amplified with the primer combinations NS1/NS8 or NS1/NS4 and NS19/NS8 or NS19/NS24 (for oligonucleotide primer sequences, see S2 Table). For Sebacinales and selected outgroup taxa, the ITS1 and ITS2 regions, including the 5.8S and the D1/D2 regions of the nuc-rDNA, were amplified with the primer combination ITS1F/NL4 and Phusion™ High-Fidelity DNA polymerase (Finnzymes Oy, Vantaa, Finland). The 28S gene of the nuc-rDNA was amplified with the primer combinations LR0R/LR9 or LR0R/LR6 and LR3R/LR9 and Phusion polymerase. In the case of negative or weak amplification of the ITS+D1/D2 or 28S region, PCRs were repeated with MangoTaq™ DNA polymerase (Bioline, Luckenwalde, Germany). To amplify the 18S, ITS, D1/D2 and 28S regions, we used PCR concentrations and cycling profiles as described by Riess *et al*. [25]. The *rpb*1 regions A and B were amplified with the primers RPB1-A/RPB1-C and MangoTaq polymerase with PCR concentration reaction indicated above and the cycling profiles of Matheny *et al*. [33]. The *atp*6 region was amplified using the primer combinations ATP6-1/ATP6-2, ATP6-3/ATP6-4, sATP6-3/ATP6-4, ATP6-SG/ATP6-4 or ATP6-F47/ATP6-R97 and MangoTaq polymerase with PCR concentration reaction indicated above and the cycling profile of Kretzer & Bruns [34].

PCR products were checked using agarose gel electrophoresis with ethidium bromide staining. If PCR products were not directly sequenceable or showed multiple bands, they were cloned using the Topo TA Cloning® Kit for Sequencing (Invitrogen, Life Technologies GmbH, Darmstadt, Germany). Colonies were used as a template for PCR with MangoTaq polymerase and M13 primers (Invitrogen).

Amplified DNA fragments were cleaned using ExoSAP-IT® reagent (USB Corporation, Cleveland, OH, USA) diluted 1:20. Purified PCR products were sequenced in both directions with 1:6 diluted BigDye® Terminator version 3.1 Cycle Sequencing Kit (Applied Biosystems, Foster City, CA, USA) on an ABI Prism 3130*xl* Genetic Analyzer (Applied Biosystems). Primers used for DNA sequencing are listed in S3 Table. Sequence fragments were assembled and verified using Sequencher version 4.10.1 (Gene Codes Corporation, Ann Arbor, MI, USA).

For Basidiomycota, a total of 99 18S, 77 28S, 98 *rpb*1 A-C and 99 *atp*6 sequences were newly generated for this study and additional sequences were downloaded from GenBank (see S1 Table). All fungal basidiomata have been either deposited in the Herbarium Tubingense or were on loan from V. Bandala, L. Ryvarden and L. Tedersoo.

### Data assembly and alignments for divergence time estimations

We assembled a sequence dataset for Basidiomycota containing 97 species representing the main clades within Agaricomycotina, 11 samples of Pucciniomycotina and Ustilaginomycotina, and three ascomycetes as outgroups (S2 Data). Sequences spanned the 18S and 28S regions, and the genes *rpb*1 (regions B–C) and *atp*6 (partial). Before concatenation of rDNA data and protein coding regions, we tested for congruency of the different regions with CADM implemented in the "ape" package version 3.1–4 in R [35]. CADM calculates concordance based on congruency of distance matrices. We calculated a pairwise distance matrix for the following regions using raw distances: 18S, 28S, *rpb*1, *atp*6. Each distance matrix needs to have the exact same number of sequences for CADM to run, which is why we had to condense our alignments to only those sequences for which data were available for all regions. For basidiomycetes, the condensed alignment contained 106 sequences (95% of original alignment). The condensed alignment was only used for congruence analyses and not for any subsequent analyses. Congruence analysis was calculated with Kendall's w and 9999 permutations.

For Sebacinales, we downloaded sequences from GenBank and UNITE databases in November 2015. We filtered the sequence data according to sequence length and available annotation of nutritional lifestyle (for more details see S3 Table). The resulting dataset 1 included 2661 ITS sequences. For further analyses, we randomly pruned this large dataset to include 693 sebacinalean ITS sequences, representing one sequence per MOTU and nutritional lifestyle. MOTU analyses were based on p-distance similarity using a threshold of 3% in combination with the single linkage algorithm performed in OPTSIL version 1.2 [36].

Ribosomal DNA sequences (18S, ITS, 28S) were aligned with MAFFT version 6.884b [37] or version 7 [38], and the L-INS-i option (for Basidiomycota) or the E-INS-i option (for Sebacinales) [39]. Nucleotide protein coding sequences of *rpb*1 and *atp*6 genes were aligned with MACSE version 0.9b1 [40]. We visually checked the alignments for quality and excluded poorly aligned regions (characters with >50% gaps for Basidiomycota with Gblocks version 0.91b [41]; and >70% gaps for Sebacinales with a custom script). For Basidiomycota, 67% of the positions were excluded from the alignment and 53% for Sebacinales.

### Maximum likelihood and divergence time estimation

Maximum likelihood (ML) phylogenies for both datasets were inferred with RAxML version 7.0.4 [42]. For the dataset with ribosomal and protein coding regions, a partitioned analysis was conducted based on each gene region. Modeltest, as implemented in the R package Phangorn [43], revealed the GTR+Γ+I model as best fit for both datasets. Therefore, the general time reversible substitution model with 1000 rapid bootstrap replicates [44] and the CAT approximation to account for evolutionary rate heterogeneity [45] was used for Basidiomycota and Sebacinales. The ML trees were used for calibration and also as starting trees for both BEAST analyses.

### Calibration points for age estimates of Sebacinales within Basidiomycota

We used three fossils to calibrate the phylogeny of Basidiomycota. Each fossil age served as a minimum constraint. *Archaeomarasmius leggettii* (Fossil #1) is a specimen preserved in amber identified as a member of the Agaricales [46]. It was used to place a minimum constraint for Agaricales. Fossil #2 is the oldest gasteromycete (72–66 million years old [47]) and was used to calibrate the Geastrales clade. Fossil #3 is a 7 million year old fungal comb produced by termites (Isoptera [48]) that was used for calibrating the split between *Tephrocybe* and *Termitomyces*.

Other basidiomycete fossils were not used because their placement within Basidiomycota is questionable. For a list of all considered fossils see S3 Data.

To place an age constraint on the split of Basidiomycota and Ascomycota, we used age estimates from previous analyses of Basidiomycota [49]. Berbee and Taylor [49] estimated the emergence of Basidiomycota between 1,489 and 452 mya depending on the placement of the ascomycetes fossil *Paleopyrenomycites* [50]. We therefore used both estimates in two separate analyses, scenario 1 and scenario 2 (see details below).

### Calibration points for age estimates of lineages within Sebacinales

We used a secondary calibration approach to obtain divergence times within Sebacinales because no fossils are available for this group. For this, we set a normal distribution as a prior, because it gives highest prior weight on the mean and is therefore suitable for secondary calibrations (BEAST 2 Tutorial; http://treethinkers.org/divergence-time-estimation-using-beast, accessed 20 November 2014).

### Divergence time estimations

Estimated divergence times, origin and phylogenetic diversification of Sebacinales were obtained with BEAST version 1.8.0 [51]. The phylogenies inferred with RAxML (S1 Fig and S2 Fig) were used as starting trees with branch lengths transformed to ages with penalized likelihood implemented in the R package Ape [35]. All groups containing calibration points were supported as monophyletic entities by the RAxML analysis and were constrained as monophyletic in the BEAST analyses. We used the general time reversible model of nucleotide substitution and chose the Yule speciation process, which is a pure birth process that specifies a constant rate of species divergence (BEAST 2 Tutorial; https://molevol.mbl.edu/wiki/images/f/ff/WHME_12_BEAST_tutorial.pdf, accessed 29 May 2014). The ‘ucld.mean’ was adjusted from 0.000001 to 10.0 in order to reflect a broad substitution range.

For Basidiomycota, we ran BEAST under two different scenarios, both with exponential distributions on all calibration nodes (including stems). Scenario 1 was a more constrained analysis using 452 mya as the divergence time for asco- and basidiomycetes. We chose a calibration method similar to [52], in that the means and offsets were chosen so that ages for calibration points would be the lower bound (offset), and the age of the next older calibration point would be within 97.5% of the prior probability. For scenario 2, 1490 mya was used as divergence time of asco- and basidiomycetes. Fossil ages were used as offset and the mean was set so that the 1490 mya would fall in 97.5% of the prior probability. For more information see XML files in S4 Data and S5 Data.

For Sebacinales, the mean ages of divergence times of Sebacinaceae and Serendipitaceae from both Basidiomycota analyses, scenario 1 and 2, were used as the mean for the normal prior. Standard deviation value and upper/lower bounds were set so that the probability of the youngest age and the oldest age of both families from both Basidiomycota analyses, scenario 1 and 2, would be within the 97.5% probability range (see S5 Data for more details).

Each analysis involved two independent runs of 100 million generations. One tree was collected per 4000 generations (burn-in was 10%). We checked the stability of the likelihood estimates, whether ESS values were acceptable and whether both runs converged on the same stationary distribution with Tracer version 1.5 [53]. LogCombiner version 1.6.2 [54] was used to combine the runs for Sebacinales. Consensus trees were created with TreeAnnotator version 1.6.2 [54]. Age estimates are reported followed by their highest posterior density (HPD) in parenthesis, which is based on 95% of all sample values. The Basidiomycota analysis was rooted using ascomycetes as outgroup and Sebacinales were rooted by the midpoint method.

### Analyses of phylogenetic structure with respect to nutritional traits in Sebacinales

The same data set as used for divergence time estimation was used to assess the phylogenetic structure and distribution of nutritional traits in Sebacinales. All nine nutritional traits for Sebacinales were included: saprobic, ectomycorrhizal, arbutoid mycorrhizal, ericoid mycorrhizal, cavendishioid mycorrhizal, orchid mycorrhizal with autotrophic or heterotrophic orchids, jungermannioid interactions with liverworts, and endophytic (S4 Table). Because heterotrophic orchids are known to exploit ectomycorrhizal fungi [19], Sebacinales forming mycorrhiza with heterotrophic orchids were pooled with ectomycorrhizal Sebacinales. In addition, a parallel analyses pooling ericoid and cavendishioid was conducted and is shown in the supplements. Two different indices were calculated using the package Picante version 1.6–2 [55] in R version 3.1.1 [56]: mean pairwise distance (MPD) between all MOTUs in each trait, and mean nearest taxon distance (MNTD) separating each MOTU in a trait from its closest relative. Therefore, the MNTD is suitable for analyzing phylogenetic clustering on the fine scale and MPD provides information about phylogenetic clustering on the large scale. Mean pairwise distance and MNTD require a distance matrix as input, which was generated directly from the ML tree in R. For both, MPD and MNTD, a null model shuffling labels across all taxa included in the matrix was used with 999 iterations and 1000 randomizations. Following these analyses, we identified one core group (main representative groups, the group that contained most sequences for a nutritional lifestyle) for each nutritional trait that was phylogenetically clustered in order to discuss divergence times for the respective traits. Note that there is more than one phylogenetically clustered group for most traits, but we limit our analysis to the largest group for each nutritional trait for simplicity.

In addition, cluster analyses of nutritional traits based on phylogenetic beta diversity were performed with R and we also gathered information on the phylogeographic provenance of each MOTU/species and included this into the Sebacinales chronogram.

## Results

### Analyses of phylogenetic structure

Sebacinales forming cavendishioid, ericoid and ectomycorrhiza (including those from achlorophyllous orchids), as well as Sebacinales associated with liverworts, were phylogenetically clustered as shown by significant MNTD and MPD values. In contrast, Sebacinales with arbutoid mycorrhiza, orchid mycorrhiza (green orchids) and endophytic interactions were not significantly clustered (Table 1).

**Table 1:** Global measures of phylogenetic clustering of Sebacinales communities based on nutritional lifestyles. Significant *P*-values (P < 0.05) for MNTD and MPD are indicated in bold. n = number of sequences (values are shown only for those categories containing ≥ 6 sequences), MNTD = mean nearest taxon distance, MPD = mean pairwise distance. MPD and MNTD are indicators of phylogenetic clustering. Pooled ericoid and cavendishioid sebacinoid sequences have also significant MNTD and MPD values.

Nutritional strategy	n	MNTD	p	MPD	p
Arbutoid	13	0.182	0.099	0.583	0.111
Cavendishioid	25	**0.063**	**0.001**	**0.358**	**0.001**
Ectomycorrhizal + Orchid (achlorophyllous)	342	**0.066**	**0.010**	**0.524**	**0.001**
Endophytic	105	0.115	0.612	0.699	0.648
Ericoid	36	**0.102**	**0.001**	**0.444**	**0.001**
Liverworts	38	**0.085**	**0.001**	**0.234**	**0.001**
Orchid (green)	132	0.113	0.907	0.756	0.999

**Fig 1:**
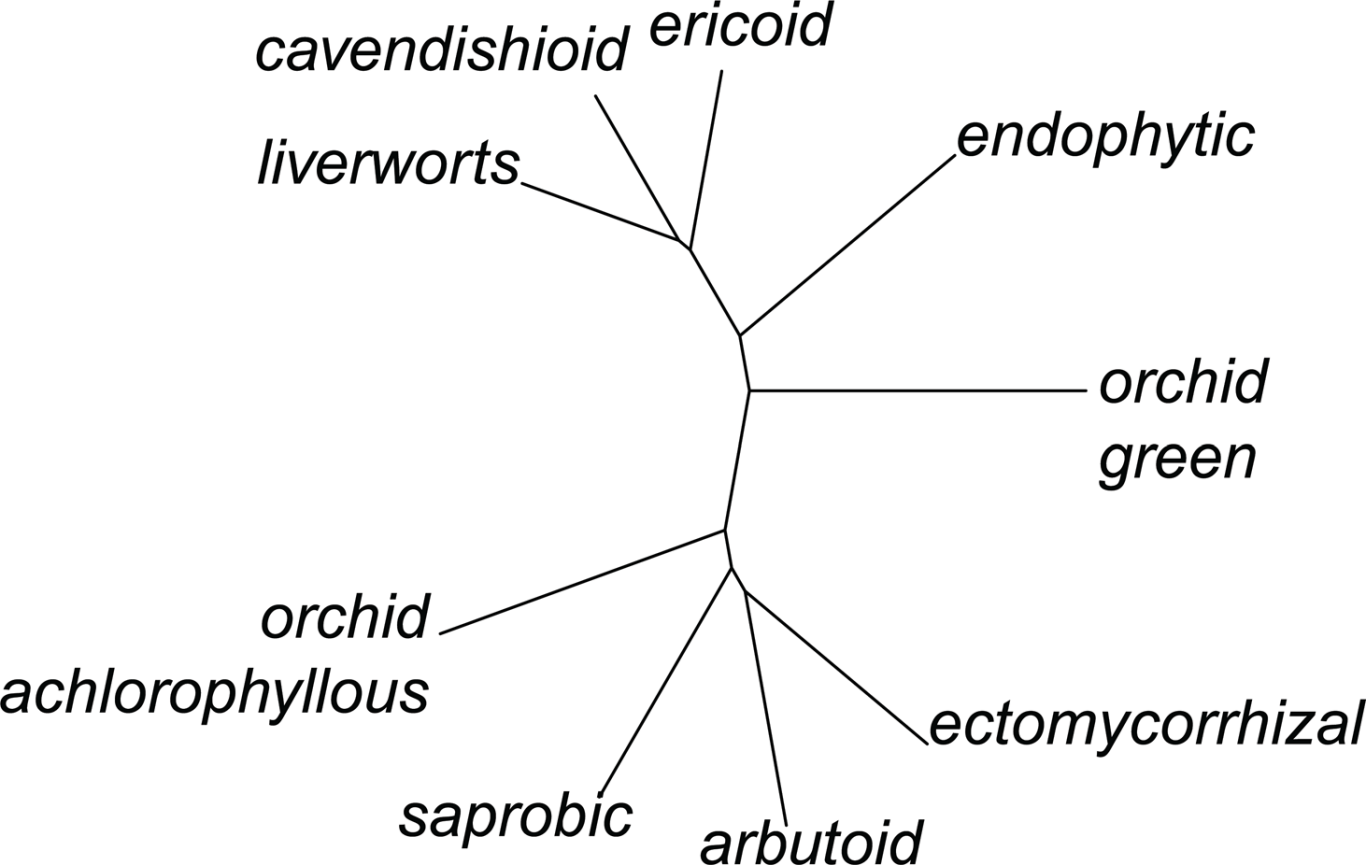
Cluster analysis of nutritional traits. The analysis was based on phylogenetic beta diversity derived from Sebacinales internal transcribed spacer (ITS) sequences.

Hierarchical clustering based on phylogenetic distances showed that ericoid, cavendishioid and jungermannioid traits are formed by closely related species of Sebacinales, as are ectomycorrhizal, saprobic and orchid mycorrhiza (achlorophyllous) traits (Fig 1). Sebacinales with arbutoid, endophytic and orchid mycorrhiza (green) traits have a more isolated position.

**Fig 2:**
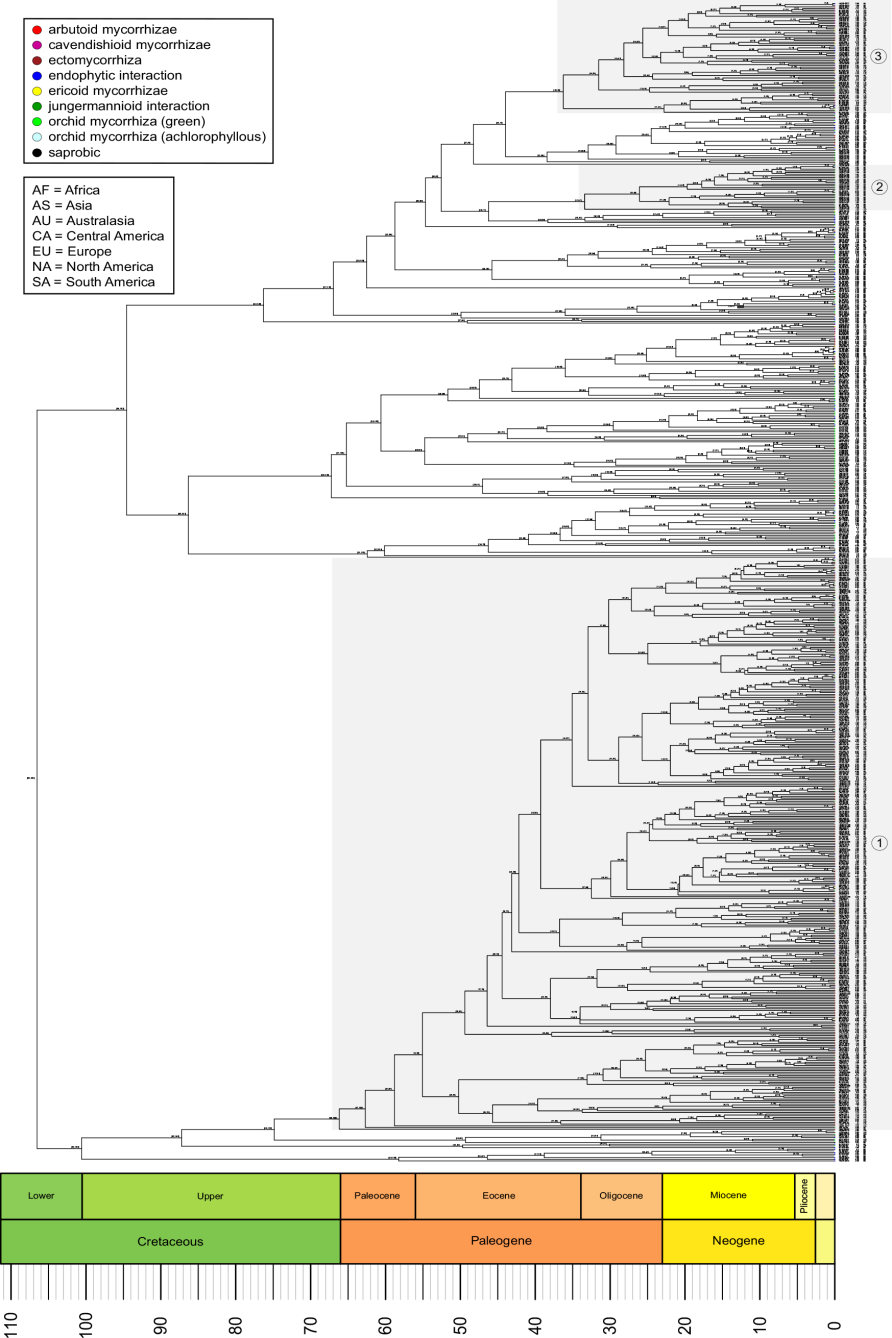
Chronogram of Sebacinales evolution. Sebacinales consensus tree obtained from the BEAST analysis based on internal transcribed spacer (ITS) alignment of 656 bp length. Core groups of major nutritional traits are shown as groups 1–3: 1) ectomycorrhiza; 2) jungermannioid; and 3) cavendishioid/ericoid. The age estimation mean for each node is given by the time scale and the 95% highest posterior density (HPD) range is drawn in square brackets next to each node. After each sequence label the assigned MOTU number and the geographical provenance are given.

Analyses of our Sebacinales dataset comprising full ITS sequences resulted in a total of 529 MOTUs (Fig 2, S4 Table). Three groups (core groups) including the main diversity of Sebacinales MOTUs with ectomycorrhizal (core group 1), jungermannioid (core group 2) and cavendishioid/ericoid nutrition (core group 3) were detected (Table 2).

**Table 2:** Main nutritional core groups found in Sebacinales. The age estimation mean for each core group is given and the 95% highest posterior density (HPD) range is drawn in square brackets.

Core group number	Nutritional core group	Support values for core group (BS/PP)	Core group age	Core group diversification start age
1	Ectomycorrhizal	90/1.0	76 [44–122]	67 [38–108]
2	Liverworts	81/0.99	47 [25–77]	34 [17–57]
3	Ericoid/cavendishioid	<50/1.0	45 [24–73]	37 [20–60]

### Divergence time estimations

Divergence time estimations calculate stem and node ages for each clade. In the following, we consider stem ages as the time a group has originated, or diverged from its sister clade, and node ages as the time a group started diversification.

**Fig 3:**
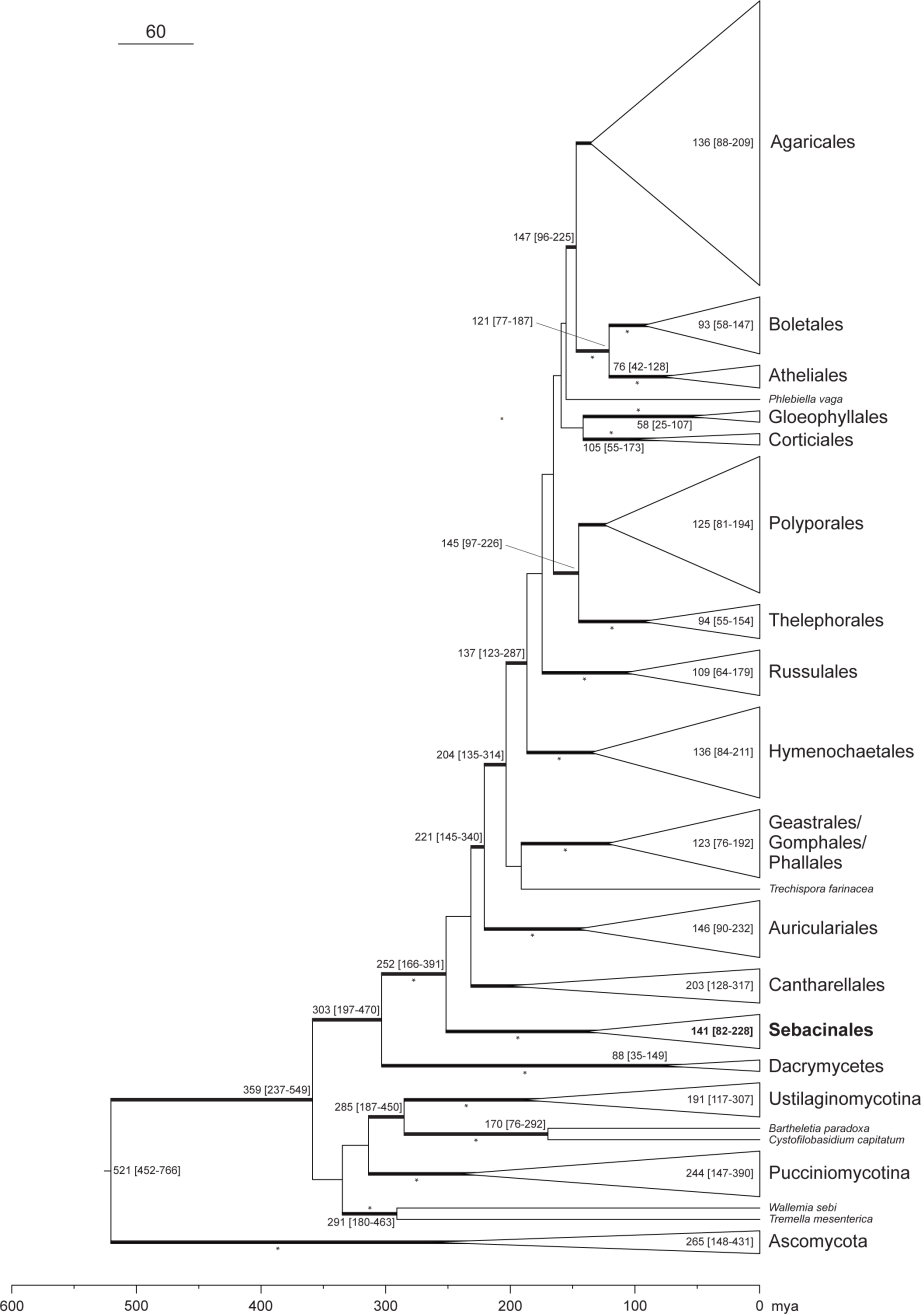
Chronogram of the main groups in Basidiomycota. The topology (scenario 2) is based on the consensus BEAST tree based on 4,436 bp of nuclear (18S, 28S, *rpb*1) and mitochondrial (*atp*6) DNA sequences. The tree was rooted with ascomycetes. For details of the species used, see S1 Table. The lines in bold indicate a posterior probability (*PP*) ≥ 0.95, and an additional asterisk denotes bootstrap values ≥ 70%. The node support and age estimation values (in mya) are only given for nodes with *PP* ≥ 0.95. The age estimation mean is followed by the 95% highest posterior density (HPD) range in square brackets.

The divergence time estimation for Basidiomycota revealed slightly different time frames for both scenarios tested. Scenario 1 estimated the divergence of Basidiomycota and Ascomycota to have occurred around 493 [452–638] mya and that Sebacinales originated around 223 [159–309] mya. Scenario 2 estimated the origin of Basidiomycota at around 521 [452–766] mya and 252 [166–391] mya for Sebacinales (Fig 3). In general, scenario 2 resulted in older age estimates than scenario 1. Within Agaricomycotina, Sebacinales represents an ancient order (Fig 3). The split of the Sebacinales in two families–Sebacinaceae and Serendipitaceae–was around 107 [68–165] mya (Fig 2). The origin of ectomycorrhiza within Sebacinaceae was likely 76 [44–122] mya, with major deep diversifications during the Eocene (core group age, approx. 67 [38–108] mya) (Fig 2: core group 1). Within Serendipitaceae, sebacinoid fungi associated with liverworts, likely originated around 47 [25–77] mya, but there are many strains outside this group that have been found in liverworts also (Fig 2: core group 2). The origin of ericoid and cavendishioid mycorrhizae is around 45 [24–73] mya, with major lineage radiations starting around 37 [20–60] mya (Fig 2: core group 3). Sebacinales associated with achlorophyllous orchids/ECM plants occur mainly within Sebacinaceae, but also in Serendipitaceae.

## Discussion

In general, age estimates for Basidiomycota and basal Ascomycota lineages calculated in our study (Fig 3) are similar to those obtained by previous analyses using a Bayesian relaxed molecular clock [52, 57, 58], and they were younger than the estimates from Heckman *et al*. [59]. The reasons for the discrepancy between Heckman *et al*.’s and other studies were previously discussed [49]. Because previous dating studies estimated the mean age split between Ascomycota and Basidiomycota around 662 mya [58] or older depending of the choice of fossil calibration points [60], we use the scenario with the oldest Ascomycota and Basidiomycota split age (scenario 2) for the presentation and discussion of our results. Only fossils with a high confidence of phylogenetic placement were included, because the uncertainty of fossil placement can lead to erroneous results [49, 61]. The fossil ages were only used as minimal age constraints, as suggested by others [62, 63], and they allowed for a high level of age uncertainty.

The split of Sebacinales into two lineages, Sebacinaceae and Serendipitaceae, occurred presumably during the late Jurassic and the early Cretaceous and was coupled with successive radiations across clades within each group (Fig 2). In the following, we will discuss only core groups for which the nutritional mode had significant MNTD and/or MPD values (see Tables 1 and 2). A major radiation during the Upper Cretaceous correlates with a shift from a saprobic or endophytic to an ectomycorrhizal (core group 1) lifestyle, at least in Sebacinaceae (Fig 2). Transition from a saprobic to ectomycorrhizal lifestyle is a relatively common ecological transition across fungal evolutionary history [64]. The ectomycorrhizal lifestyle in the /sebacina lineage [30] (corresponds to our core group 1) probably arose during the Upper Cretaceous, 76 [44–122] mya, and was retained throughout subsequent diversifications (Fig 2). Our results support an older origin for the ectomycorrhizal lifestyle than those of Tedersoo *et al*. [30], who estimated an age of 45–58 mya for the ectomycorrhiza (/sebacina) lineage. The discrepancy in divergence times between our and the Tedersoo et al. [30] paper is not surprising, because Tedersoo and coauthors chose a different, more constricted calibration method for their analyses. Tedersoo et al. [30] used a normal distributed age prior on the tree height with a mean of 430 myr and a standard deviation of 25 myr, which does not account for a much earlier age of Basidiomycota. However, the placement of the ascomycete fossil *Paleopyrenomycites devonicus* [65] provides a solid minimum age constraint for Basidiomycota at 452 myr [50] and depending on its placement, could even push the origin of Basidiomycota further back to 1489 myr [50]. We, therefore, think that the divergence times estimated by Tedersoo et al. [30] for ectomycorrhizal Sebacinales are too young and likely a result of their constricted calibration method.

Sebacinales has a relatively ancient origin within Basidiomycota evolution, but the origin of the ectomycorrhizal lifestyle is in line with other fungal groups, as Agaricales and Tuberaceae [66, 67]. The age estimation for the change from saprobic to ectomycorrhizal nutrition in Sebacinales coincides with that observed in Serpulaceae [68]. The mean age of origin for ectomycorrhizae (76 mya) in Sebacinaceae is younger than the emergence of Fagales (~84 mya [69]; ~100 mya [70]; or ~110 mya [71]), but the confidence intervals [44–122 mya] show that it is possible for both groups to have emerged around the same time. At approximately 128 mya [72], Pinaceae however, are likely much older than ectomycorrhizal Sebacinales, because the oldest ectomycorrhizal fossil is from the middle Eocene [73]. As in previous studies [23, 24], our analyses support close relationships between Sebacinales involved in ectomycorrhizal and achlorophyllous orchid lifestyles. Sebacinales ecology becomes more complex if we consider that the same MOTUs/species can be involved in more than one nutritional lifestyle as previously reported [19, 24]. Within Serendipitaceae, the core group of Sebacinales associated with liverworts (core group 2) diversified during the Oligocene and is younger than radiation events of their hosts [74]. However, the origin of the jungermannioid nutrition mode and its significant phylogenetic structure should be carefully interpreted due to the small number of samples available for analysis, which were also restricted in terms of distribution. Major diversification for ericoid and cavendishioid mycorrhizal strategies in Serendipitaceae (core group 3) began during the Eocene/Oligocene period, which coincides with diversification events of Vaccinieae (~ 46 mya), *Gaultheria* (~ 29 mya) and *Erica* (~ 29 mya) [75].

Although Sebacinales represents a relatively old order in Basidiomycota evolution, the age estimations of sebacinoid fungi show that geographically mixed clades are relatively young, e.g. Sebacinales from Europe/Africa (MOTU 196) and South America (MOTU 181) or from Africa (MOTU 80) and South America (MOTU 176) belong to clades younger than 12 mya (Fig 2; S4 Table). Our age estimates suggest that vicariance is unlikely to be a reason for the geographic pattern of the extant Sebacinales (252 [166–391] mya, scenario 2), because Laurasia and Gondwanaland separated about 180 mya [76]. Human-mediated activities (e.g. reforestation or development) might explain how Sebacinales could have spread in terrestrial environments, which agrees with the hypothesis of multiple recent dispersal events as postulated [30]. However, this is unlikely the only reason, because some distantly related sequences occurred in pristine habitats and were not associated with plants of economic value [77].

It is difficult to infer the main factors shaping modern distribution and phylogenetic structure of Sebacinales at a global scale, mainly due to lack of fossil records, sampling [30] and nutrition trait unspecificity [78]. In addition, most of the available sequences for Sebacinales comprise either ITS or the 28S rDNA region. However, each region alone is not informative enough to resolve the backbone of the phylogeny [25]. Therefore, further investigations including more homogenous sampling of Sebacinales from plant families covering their distribution ranges, as well as the use of more genetically informative DNA regions (e.g. ITS+LSU D1/D2 regions of the rDNA), could be helpful in gaining a stronger phylogenetic signal of the modern global patterns of distribution and ecological specialisation of Sebacinales.

## Supporting Information

S1 Table Basidiomycota specimens used in this study. The species names, collection numbers, herbarium vouchers or strain identifiers and the GenBank accession numbers are given. Sequences newly generated for this study are indicated in bold.
(XLS)

S2 Table List of primers with nucleotide sequences used in this study. Primers marked with asterisks were only used for DNA sequencing.
(XLS)

S3 Table Sebacinales ITS sequences from GenBank and UNITE databases. Sequences were retrieved from GenBank using the ("Sebacina"[Organism] OR "Sebacinaceae"[Organism] OR "Sebacinales"[Organism]) AND ("internal transcribed spacer 1"[All Fields] OR "ITS"[All Fields]) AND "internal transcribed spacer 2"[All Fields] search criteria. The following criteria were used to filter the ITS sequence dataset: a) sequences with a length < 500 bp, b) aligned sequences with less than 60% of longest sequence, c) sequences generated from soil, d) fruit bodies, hyphae or mycelia with unknown ecology, e) without ecological information and f) sequences incorrectly labelled as Sebacinales.
(XLS)

S4 Table Table containing MOTU designations, nutrition strategies, geographic distributions, and GenBank accession numbers of sequences used for analysis in R. (XLS)

S1 Data ITS alignment of Sebacinales used for age estimation and assessment of phylogenetic structure. (FASTA)

S2 Data A concatenated sequence alignment of the Basidiomycota dataset (i). The alignment comprises 18S, 28S, *rpb*1 and *atp*6 sequences with a length of 4,438bp.
(NEX)

S3 Data This file contains information about the fossil calibration approach used for Basidiomycota. Including a table of all Basidiomycota fossils with species name (if available), times, epochs, citations and calibration usages.
(DOC)

S4 Data This file contains information about the priors and parameters used in BEAST to obtain Basidiomycota age estimates. (TXT)

S5 Data This file contains information about the priors and parameters used in BEAST to obtain Sebacinales age estimates. (XML)

S1 Fig A maximum likelihood (ML) tree of Basidiomycota with calibration points. Bootstrap values ≥ 70% are given.
(PDF)

S2 Fig A maximum likelihood (ML) tree of Sebacinales with bootstrap values. (PDF)
