# Supplementary material for: Divergence Times and Phylogenetic Patterns of Sebacinales, a Highly Diverse and Widespread Fungal Lineage
Source: PLoS One. 2016 Mar 3;11(3):e0149531. doi: 10.1371/journal.pone.0149531 (PMC4795679; doi:10.1371/journal.pone.0149531)
Supplement: S3 Data — Including a table of all Basidiomycota fossils with species name (if available), times, epochs, citations and calibration usages. (DOC) [file pone.0149531.s007.doc]

**Overview of fossils in Basidiomycota**

Ages of fossils in bold were used as minimum age constraints to calibrate the BEAST analyses for basidiomycetes. Many fossils were not included because their phylogenetic position was not certain or the fossils were too young. Ages are in million years.

| **Species** | **Affiliation** | **From** | **To** | **Epoch** | **Minimum age constraint for** | **Citation** |
| --- | --- | --- | --- | --- | --- | --- |
| not identified to species level | Basidiomycota | 541 | 485.4 | Cambrian | Basidiomycota | Brunel et al. 1984 |
| not identified to species level | Basidiomycota | 541 | 485.4 | Cambrian | Basidiomycota | Brunel et al. 1985 |
| not identified to species level | Basidiomycota | 382.7 | 358.9 | Upper Devonian | Basidiomycota | Stubblefield et al. 1985 |
| not identified to species level | Basidiomycota | 330 |  | Middle to upper Mississippian | Basidiomycota | Krings et al. 2011 |
| *Palaeancistrus martinii* | Basidiomycota | 315.2 | 307 | Middle Pennsylvanian | Basidiomycota | Dennis 1970 |
| not identified to species level | Basidiomycota | 252.17 | 201.3 | Triassic | Basidiomycota | Stubblefield & Taylor 1986 |
| not identified to species level | Basidiomycota | 237 | 201.3 | Upper Triassic | Basidiomycota | Creber & Ash 1990 |
| *Paleaeocigaracites antiquus* | Basidiomycota | 145 | 100.5 | Lower Creataceous | Basidiomycota | Poinar & Buckley 2007 |
| *Quatsinoporites cranhamii* | Polyporales or Boletales (?) | 118 | 113 | Barremian | Polyporales (?) | Smith et al. 2004 |
| *Paleoclavariaceae* | Basidiomycota | 100 |  | Upper Creataceous | Basidiomycota | Poinar & Brown 2003 |
| ***Archaeomarasmius leggetti*** | **Tricholomataceae** | **94** | **90** | **Turonian** | **Agaricales** | **Hibbett et al. 1997** |
| *Lithopolyporales zeerabadensis* | Polyporales | 72.1 | 66 | Maastrichtian Age | Boletales or Polyporales | Kar et al. 2003 |
| ***Gastroidea lobata*** | **Gasteromycetes** | **72.1** | **66** | **Maastrichtian Age** | **Gasteromycetes** | **Krassilov & Makulbekov 2003** |
| *Trametites eocenicus* | Polyporaceae | 56 | 33.9 | Bohemian Eocene | Boletales or Polyporales | Knobloch & Kotlaba 1994 |
| *Paleaocybe striata* | Basidiomycota | 56 | 33.9 | Eocene | Basidiomycota | Doerfelt & Striebich 2000 |
| *Appianoporites vancouverensis* | Hymenochaetales | 56 | 33.9 | Eocene | Boletales or Polyporales | Smith et al. 2004 |
| not identified to species level | Basidiomycota | 50 |  | Middle Eocene | Basidiomycota | LePage et al. 1994 |
| *Suillus* (?)*, Rhizopogon* (?) | Basidiomycota | 50 |  | Middle Eocene | Suillus (?) or Rhizopogon (?) | LePage et al. 1997 |
| *Coprinites dominicana* | Coprinaceae | 40 | 35 | Upper Eocene | Phylogenetic placement uncertain | Poinar & Singer 1990 |
| not identified to species level | Basidiomycota | 33.9 | 23.03 | Oligocene | Basidiomycota | Legrand 2012 |
| *Geastrum tepexensis* | Gasteraceae | 23.03 | 1.806 | Miocene - Lower Pleistocene | Gasteraceae | Magallon-Puebla & Cevallos-Ferriz 1993 |
| *Ganodermites libycus* | Polyporales | 23.03 | 2.588 | Neogene | Boletales or Polyporales | Fleischmann et al. 2007 |
| *Protomycena electra* | Tricholomataceae | 23.03 | 5.333 | Miocene | Phylogenetic placement uncertain | Hibbett et al. 1997 |
| *Aureofungus yaniguanensis* | Euagarics Clade | 20 | 15 | Miocene | Phylogenetic placement uncertain | Hibbett et al. 2003 |
| ***Termitomyces*** | **Basidiomycota** | **7** |  | **Miocene** | **Termitomyces** | **Duringer et al. 2006** |
| not identified to species level | Polyporaceae | 3.6 | 2.588 | Upper Pliocene | Boletales or Polyporales | Lutz 1993 |
| not identified to species level | Polyporales | 2.588 | 0.012 | Quarternary | Boletales or Polyporales | Kreisel & Ansorge 2009 |

For cases in which absolute ages for fossils were not given by authors, we used the International Chronostratigraphic Chart (www.stratigraphy.org/ICSchart/ChronostratChart2013-01.pdf, accessed on 28.02.2014) to determine absolute ages.

**References**

Aanen DK, Ros VI, de Fine Licht HH, Mitchell J, de Beer ZW, Slippers B, Rouland-LeFèvre C, Boomsma JJ (2007) Patterns of interaction specificity of fungus-growing termites and *Termitomyces* symbionts in South Africa. *BMC Evolutionary Biology*, **7**, 115.

Brunel M, D’Albissin C, Locquin M (1984) Cambrian age determination by the discovery of paleobasidiosporae in the magnesite-bearing carbonated series underlying the Great Himalayan thrust main central thrust-eastern Nepal. *Geobios*, **17**, 595–610.

Brunel M, D’Albissin C, Locquin M (1985) The Cambrian age of magnesites from east Nepal as determined through the discovery of paleobasidiospores. *Journal of the Geological Society of India*, **26**, 255–260.

Creber TG, Ash RS (1990) Evidence of widespread fungal attack on upper Triassic tress in the Southwestern USA. *Review of Palaeobotany and Palynology*, **63**, 189–196.

Dennis RL (1970) A Middle Pennsylvanian Basidiomycete Mycelium with Clamp Connections. *Mycologia*, **62**, 578–584.

Doerfelt H, Striebich B (2000) *Palaeocybe striata*, a new fossil fungus in amber from the Tertiary. *Zeitschrift fuer Mykologie*, **66**, 27–34.

Duringer P, Schuster M, Genise JF, Likius A, Mackaye HT, Vignaud P, Brunet M (2006) The first fossil fungus gardens of Isoptera: oldest evidence of symbiotic termite fungiculture (Miocene, Chad basin). *Naturwissenschaften*, **93**, 610–615.

Fleischmann A, Krings M, Mayr H, Agerer R (2007) Structurally preserved polypores from the Neogene of North Africa: Ganodermites libycus gen. et sp. nov. (Polyporales, Ganodermataceae). *Review of Palaeobotany and Palynology*, **145**, 159–172.

Garnica S, Weiß M, Walther G, Oberwinkler F (2007) Reconstructing the evolution of agarics from nuclear gene sequences and basidiospore ultrastructure. *Mycological Research*, **111**, 1019–1029.

Hibbett D, Grimaldi D, Donoghue M (1997) Fossil mushrooms from Miocene and Cretaceous ambers and the evolution of Homobasidiomycetes. *American Journal of Botany*, **84**, 981–981.

Hibbett DS, Binder M, Bischoff JF, Blackwell M, Cannon PF, Eriksson OE, Huhndorf S, James T, Kirk PM, Lücking R, *et al.* (2007) A higher-level phylogenetic classification of the Fungi. *Mycological Research*, **111**, 509–547.

Hofstetter V, Clémencon H, Vilgalys R, Moncalvo JM (2002) Phylogenetic analyses of the *Lyophyllaceae* (Agaricales, Basidiomycota) based on nuclear and mitochondrial rRNA sequences. *Mycological Research*, **106**, 1043–1059.

Kar RK, Sharma N, Agarwal A, Kar R (2003) Occurrence of fossil-wood rotters (polyporales) from the Lameta Formation (Maastrichtian), India. *Current Science (Bangalore)*, **85**, 37–40.

Knobloch E, Kotlaba F (1994) Trametites eocenicus, a new fossil polypore from the Bohemian Eocene. *Czech Mycology*, **47**, 207–213.

Krassilov VA, Makulbekov NM (2003) The first finding of gasteromycetes in the Cretaceous of Mongolia. *Paleontologicheskii Zhurnal*, **37**, 439-442.

Kreisel H, Ansorge J (2009) Subfossil polypores from the Quaternary of the Vorpommern region. *Zeitschrift fuer Mykologie*, **75**, 33–50.

Krings M, Dotzler N, Galtier J, Taylor TN (2011) Oldest fossil basidiomycete clamp connections. *Mycoscience*, **52**, 18–23.

Legrand P-G (2012) Oligocene fossil oak woods, Quercoxylon lecointrei GAZEAU & KOENIGUER, decayed by a pocket-rot fungus. *Palaeontographica Abteilung B*, **289**, 27–41.

LePage BA, Currah RS, Stockey RA (1994) The Fossil Fungi of the Princeton Chert. *International Journal of Plant Sciences*, **155**, 828–836.

Lutz AI (1993) Two Pliocene xylophilous Basidiomycetes (Polyporaceae) of Entre Rios, Argentina. *Ameghiniana*, **30**, 419–422.

Magallon-Puebla S, Cevallos-Ferriz SRS (1993) A Fossil Earthstar (Geasteraceae; Gasteromycetes) from the Late Cenozoic of Puebla, Mexico. *American Journal of Botany*, **80**, 1162–1167.

Moncalvo JM, Vilgalys R, Readhead SA, Johnson JE, James TY, Aime MC, Hofstetter V, Verduin SJW, Larsson E, Baroni TJ, *et al.* (2002) One hundred and seventeen clades od euagarics. *Molecular Phylogenetics and Evolution*, **23**, 357–400.

Poinar GO, Brown AE (2003) A non-gilled hymenomycete in Cretaceous amber. *Mycological Research*, **107**, 763–768.

Poinar GO, Buckley R (2007) Evidence of mycoparasitism and hypermycoparasitism in Early Cretaceous amber. *Mycological Research*, **111**, 503–506.

Poinar GO, Singer R (1990) Upper eocene gilled mushroom from the dominican republic. *Science*, **248**, 1099.

Smith SY, Currah RS, Stockey RA (2004) Cretaceous and Eocene Poroid Hymenophores from Vancouver Island, British Columbia. *Mycologia*, **96**, 180–186.

Stubblefield SP, Taylor TN (1986) Wood Decay in Silicified Gymnosperms from Antarctica. *Botanical Gazette*, **147**, 116–125.

Taylor TN, Hass H, Kerp H (1999) The oldest fossil ascomycetes. *Nature*, **399**, 648–648.
