# Supplementary material for: Divergence Times and Phylogenetic Patterns of Sebacinales, a Highly Diverse and Widespread Fungal Lineage
Source: PLoS One. 2016 Mar 3;11(3):e0149531. doi: 10.1371/journal.pone.0149531 (PMC4795679; doi:10.1371/journal.pone.0149531)
Supplement: S1 Fig — Bootstrap values ≥ 70% are given. (PDF) [file pone.0149531.s010.pdf]

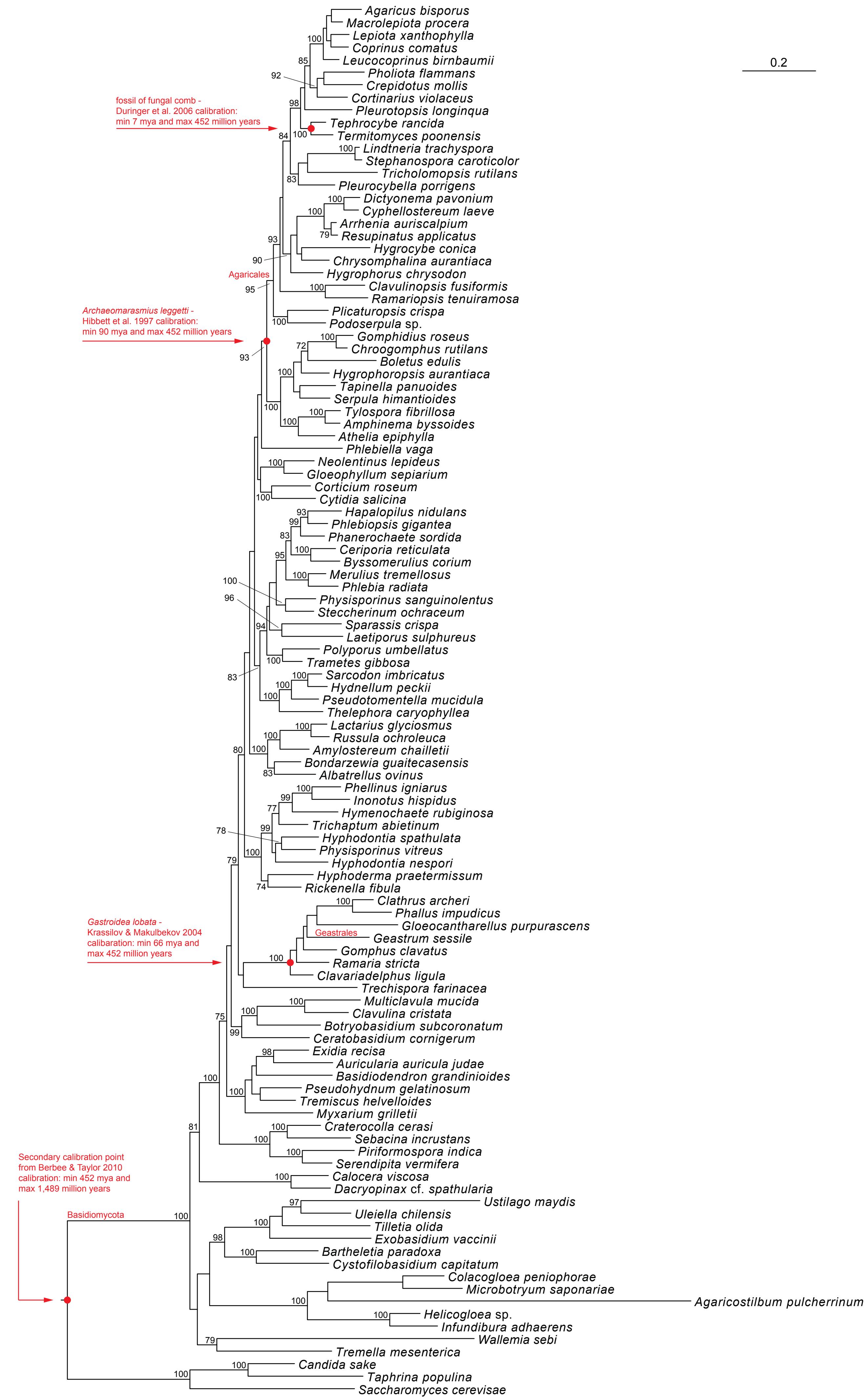

0.2
Agaricus bisporus
Macrolepiota procera
Lepiota xanthophylla
Coprinus comatus
Leucocoprinus birnbaumii
Pholiota flammans
Crepidotus mollis
Cortinarius violaceus
Pleurotopsis longinqua
fossil of fungal comb -
Duringer et al. 2006 calibration:
min 7 mya and max 452 million years
Tephrocybe rancida
Termitomyces poonensis
Lindtneria trachyspora
Stephanospora caroticolor
Tricholomopsis rutilans
Pleurocybella porrigens
Dictyonema pavonium
Cyphellostereum laeve
Arrhenia auriscalpium
Resupinatus applicatus
Hygrocybe conica
Chrysomphalina aurantiaca
Hygrophorus chrysodon
Agaricales
Clavulinopsis fusiformis
Ramariopsis tenuiramosa
Archaeomarasmius leggetti -
Hibbett et al. 1997 calibration:
min 90 mya and max 452 million years
Plicaturopsis crispa
Podoserpula sp.
Gomphidius roseus
Chroogomphus rutilans
Boletus edulis
Hygrophoropsis aurantiaca
Tapinella panuoides
Serpula himantioides
Tylospora fibrillosa
Amphinema byssoides
Athelia epiphylla
Phlebiella vaga
Neolentinus lepideus
Gloeophyllum sepiarium
Corticium roseum
Cytidia salicina
Hapalopilus nidulans
Phlebiopsis gigantea
Phanerochaete sordida
Ceriporia reticulata
Byssomerulius corium
Merulius tremellosus
Phlebia radiata
Physisporinus sanguinolentus
Steccherinum ochraceum
Sparassis crispa
Laetiporus sulphureus
Polyporus umbellatus
Trametes gibbosa
Sarcodon imbricatus
Hydnellum peckii
Pseudotomentella mucidula
Thelephora caryophyllea
Lactarius glyciosmus
Russula ochroleuca
Amylostereum chailletii
Bondarzewia guaitecasensis
Albatrellus ovinus
Phellinus igniarus
Inonotus hispidus
Hymenochaete rubiginosa
Trichaptum abietinum
Hyphodontia spathulata
Physisporinus vitreus
Hyphodontia nespori
Hyphoderma praetermissum
Rickenella fibula
Clathrus archeri
Phallus impudicus
Gloeocantharellus purpurascens
Geastrales
Geastrum sessile
Gomphus clavatus
Gastroidea lobata -
Krassilov & Makulbekov 2004
calibaration: min 66 mya and
max 452 million years
Ramaria stricta
Clavariadelphus ligula
Trechispora farinacea
Multiclavula mucida
Clavulina cristata
Botryobasidium subcoronatum
Ceratobasidium cornigerum
Exidia recisa
Auricularia auricula judae
Basidiodendron grandinioides
Pseudohydnum gelatinosum
Tremiscus helvelloides
Myxarium grilletii
Craterocolla cerasi
Sebacina incrustans
Piriformospora indica
Serendipita vermifera
Secondary calibration point
from Berbee & Taylor 2010
calibration: min 452 mya and
max 1,489 million years
Calocera viscosa
Dacryopinax cf. spathularia
Basidiomycota
Ustilago maydis
Uleiella chilensis
Tilletia olida
Exobasidium vaccinii
Bartheletia paradoxa
Cystofilobasidium capitatum
Colacogloea peniophorae
Microbotryum saponariae
Agaricostilbum pulcherrinum
Helicogloea sp.
Infundibura adhaerens
Wallemia sebi
Tremella mesenterica
Candida sake
Taphrina populina
Saccharomyces cerevisae
