# Supplementary material for: Divergence Times and Phylogenetic Patterns of Sebacinales, a Highly Diverse and Widespread Fungal Lineage
Source: PLoS One. 2016 Mar 3;11(3):e0149531. doi: 10.1371/journal.pone.0149531 (PMC4795679; doi:10.1371/journal.pone.0149531)

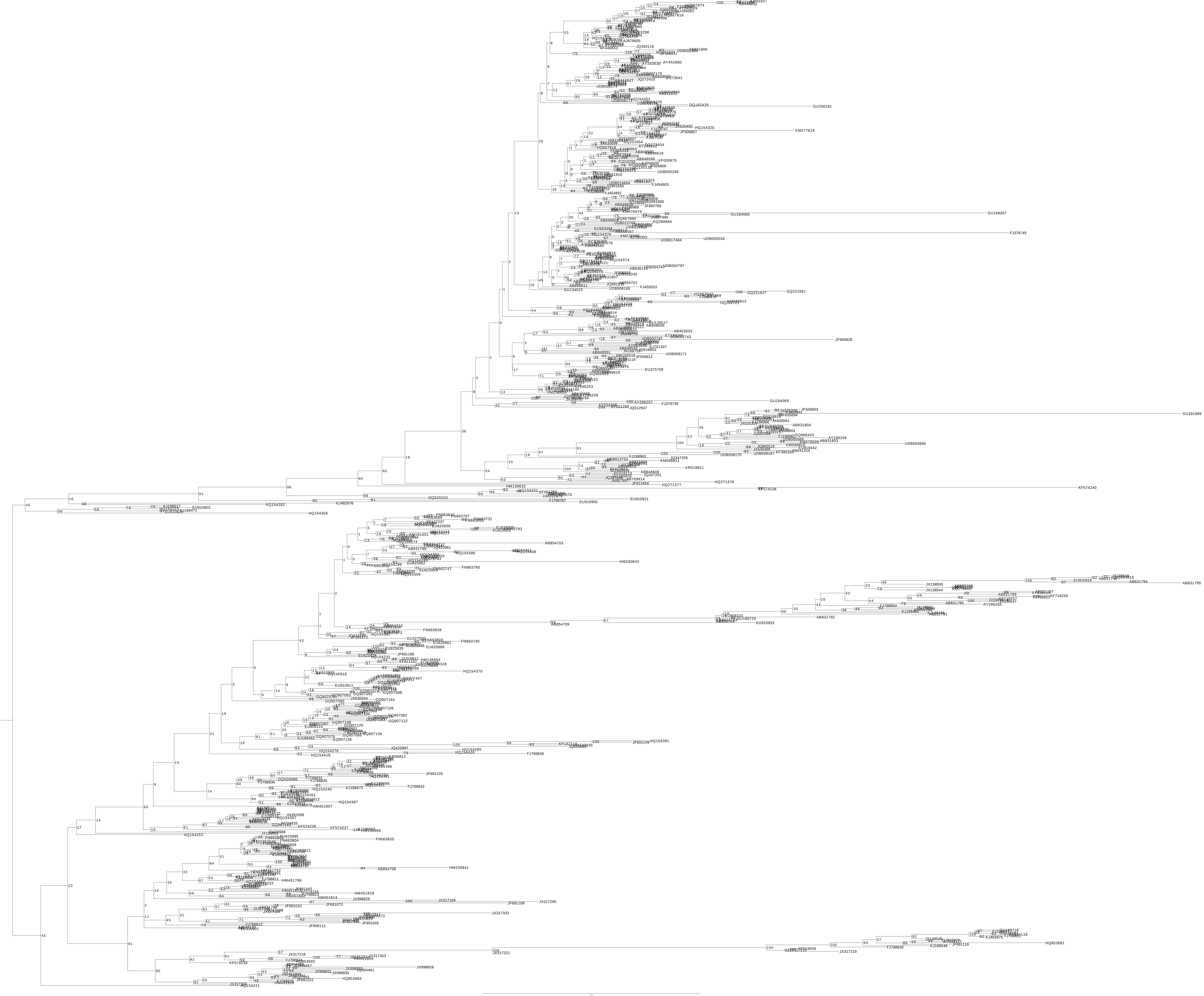

Supplement: S2 Fig — (PDF) [file pone.0149531.s011.pdf]
